# The role of molecular biomarkers in the diagnosis, prognosis, and treatment stratification of oral squamous cell carcinoma: A comprehensive review

**DOI:** 10.1016/j.jlb.2025.100285

**Published:** 2025-01-04

**Authors:** Saravanan Ravindran, Srinivasan Ranganathan, Karthikeyan R, Nandini J, Shanmugarathinam A, Senthil Kumar Kannan, Durga Prasad K, Jalaiah Marri, Rajaganapathi K

**Affiliations:** a Faculty of Pharmacy, Bharath Institute of Higher Education and Research, Chennai, 73, India; b School of Pharmacy, Sri Balaji Vidyapeeth, SBV Campus, Pillayarkuppam, Puducherry, India; c Saveetha college of pharmacy, Saveetha institute of medical and technical sciences, Chennai, 602105, India; d Department of Pharmaceutical Technology, University College of Engineering, Bharathidasan Institute of Technology Campus, Anna University, Tiruchirappalli, 620024, India; e Department of Pharmaceutics Karpagam college of pharmacy, Othakkalmandapam, Coimbatore, 32, India; f K.V.S.R. Siddhartha College of Pharmaceutical Sciences, Vijayawada, Andhra Pradesh, 520008, India; g QIS College of Pharmacy, Vengamukkapalem, Ongole, Andhra Pradesh, 523272, India

**Keywords:** Oral squamous cell carcinoma (OSCC), Molecular biomarkers, Diagnosis, Prognosis, Treatment stratification

## Abstract

One of the most common cancers targeting the area of the head and neck is oral squamous cell carcinoma (OSCC), carrying a heavy global health cost. With a high incidence of metastasis and recurrence, the outlook for OSCC remains dismal despite advancements in treatment. This has sparked an investigation into molecular biomarkers, which have the potential to improve early diagnosis, forecast patient outcomes, and direct therapeutic approaches. An extensive summary of the function of molecular biomarkers in OSCC diagnosis, prognosis, and medical care stratification is given in this article. Complex genetic mutations, epigenetic changes, and dysregulated signalling pathways are all part of the aetiology of OSCC. *Tumor protein p53* (Tp53), *Epidermal growth factor receptor* (EGFR-targeted), *Cyclin D1* (CCND1), and *Human papilloma virus* (HPV) status are examples of molecular biomarkers that have demonstrated potential in recognising disease at an early stage and identifying malignant changes. The non-invasive detection capabilities of diagnostic biomarkers such as salivary proteins, circulating tumour DNA (ctDNA), and *microRNAs* are being explored more and more because they may provide early intervention and better patient outcomes. Prognostically, tumour aggressiveness, recurrence risk, and overall survival have all been linked to biomarkers such as *matrix metalloproteinases* (MMPs), *E-cadherin*, and different cytokines. Furthermore, immune checkpoints such as *cytotoxic T-lymphocyte-associated protein 4* (CTLA-4) and *programmed death-ligand 1* (PD-L1) are becoming recognised as important markers of the tumour microenvironment's function in the course of the disease and its reaction to immunotherapy. The significance of biomarkers in personalised medicine has been further highlighted by the recognition of subgroups with elevated risk that might gain benefit from more aggressive treatment options thanks to the genetic profiling of OSCC. Predictive biomarkers are essential for therapy classification because they allow therapeutic regimens to be tailored. For example, (K*irsten rat sarcoma viral oncogene homologous)* KRAS mutations and EGFR expression influence the effectiveness of targeted therapies, and the existence of specific epigenetic markers influences choices about radiation or chemotherapy. It is expected that the incorporation of multi-omics techniques, which integrate transcriptome, proteome, and genomic data, will improve these tactics and increase accuracy in OSCC treatment. Molecular indicators have the potential to significantly improve the medical treatment of ovarian cancer. Better patient outcomes will eventually result from earlier identification, more precise prognostication, and individualised therapy regimens made possible by advancements in biomarker research. For these biomarkers to be widely used, further research must be done on verifying them and incorporating them into standard clinical practice.

## Highlights

• Treatment stratification of oral squamous cell carcinomas using molecular biomarkers.
• Predictive biomarkers for chemotherapy and radiotherapy responses.
• Targeted therapies and the role of biomarkers in treatment selection.
• Current trends and future challenges in biomarkers for oral squamous cell carcinoma.
• Biomarkers for oral squamous cell carcinomas immunotherapy.

## 1 Introduction

**Fig. 1:**
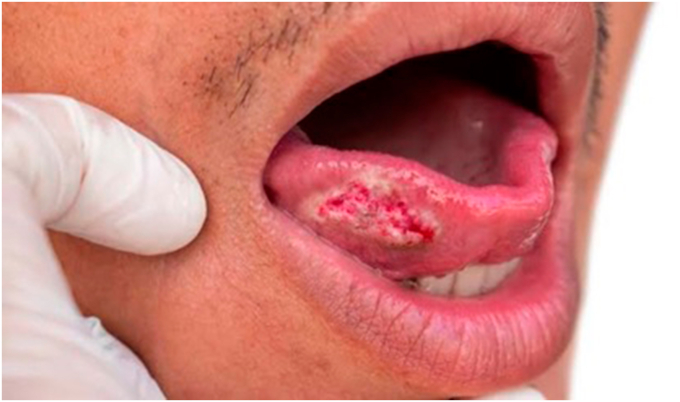
Oral squamous cell carcinoma Mouth cancer.

About ninety percent of all oral malignancies are oral squamous cell carcinomas (OSCCs), one of the most common types of head and neck cancer. It is still a major global public health concern, especially in areas where alcohol and tobacco use are common. Patients with OSCC still have a dismal prognosis despite significant improvements in diagnosis and therapy because of high rates of metastasis, recurrence, and treatment resistance. Due mostly to late-stage diagnoses, the five-year survival rate of patients with OSCC has plateaued at 50–60 %, highlighting the need for more accurate and dependable diagnostic and prognostic instruments. A plethora of genetic and epigenetic changes, as well as disturbances in important signalling pathways that drive tumour start, make up the extremely complex molecular landscape of OSCC [1]. The investigation of biological markers as instruments for enhancing the medical treatment of OSCC has arisen because of this intricacy. Based on their function in clinical practice, biomarkers—measurable indicators of biological states or conditions—can be categorised as diagnostic, prognostic, or predictive [2] (see Fig. 1).

Improved survival rates and efficient treatment for OSCC depend on early diagnosis. Nevertheless, the disease is frequently discovered at an advanced stage by the existing diagnostic techniques, which depend on clinical examination and histological study. With the potential to provide non-invasive or minimally invasive screening techniques, molecular biomarkers have become increasingly attractive as early detection tools. Important molecular indicators, including *Human papilloma virus* (HPV) status, *Epidermal growth factor receptor* (EGFR-targeted), *Tumor protein p53* (Tp53), and *Cyclin D1* (CCND1), have shown great promise in early OSCC detection. One of the most frequent genetic changes in OSCC is a mutation in the p53 gene, which is linked to the development of malignant states from precancerous lesions. In a similar vein, poor prognosis and tumour development have been linked to overexpression of EGFR and Cyclin D1 [3]. Salivary proteins, *circulating tumour DNA* (ctDNA), and *microRNAs* (miRNAs) are examples of non-invasive diagnostic biomarkers that are being investigated more and more for their potential to identify OSCC early on. Particularly, salivary diagnostics provide an easy and non-invasive way to screen for OSCC early on; multiple studies have shown the value of various salivary proteins and miRNAs as biomarkers. Another area of ongoing research is the detection of ctDNA, which represents the genetic profile of the tumour, in bodily fluids. This technique has the potential to detect minimal residual illness and track tumour dynamics [4]. Prognostic biomarkers help clinicians anticipate outcomes including tumour aggressive behaviour, repetition, and overall survival by giving vital information about the probable progression of the disease. Finding trustworthy prognostic biomarkers for OSCC is crucial for risk assessment and treatment plan optimisation. E-cadherin, different cytokines, and *matrix metalloproteinases* (MMPs) are some of the most researched predictive indicators. MMPs in particular, MMP-2 and MMP-9—have been connected to increased tumour invasiveness and metastasis and are implicated in the destruction of the extracellular matrix. Another sign linked to the *epithelial-to-mesenchymal transition* (EMT), a critical step in the development of cancer that raises the risk of metastasis, is the loss of *E-cadherin* expression [5]. It is anticipated that the incorporation of multi-omics techniques, which integrate transcriptome, proteome, and genetic data, will enhance OSCC therapy plans. These methods can offer a thorough understanding of the molecular changes causing the illness, making it possible to find new therapeutic targets and create combination treatments that work better together. Immunotherapy is being investigated for OSCC; immune-related biomarkers such as *programmed death-ligand 1* (PD-L1) are being used to identify individuals who might benefit from such treatments. Immunotherapy has demonstrated promise in other malignancies [[6], [7], [8]].

The intricacy of OSCC, which is caused by a multitude of genetic and epigenetic changes, highlights the potential of biomarkers that are to revolutionise the diagnosis, prognosis, and treatment of this condition. Molecular biomarkers provide potential for early intervention since they can identify modest changes in biology at the level of molecules even prior to the onset of clinical symptoms. These biomarkers also shed light on tumour behaviour, which helps forecast how the disease will progress and how well a particular treatment would work. Using molecular biomarkers in clinical practice has the potential to improve patient outcomes by allowing for the customisation of personalised treatment regimens as precision medicine advances [9]. In light of the recent explosion in biomarker research and the mounting evidence for their potential therapeutic applications, a thorough evaluation of the body of knowledge is imperatively needed. This study provides a comprehensive overview of the most important molecular indicators in OSCC with the goal of bridging the gap between research and clinical practice. It will investigate the ways in which these biomarkers can improve prognosis models, facilitate early identification, and direct therapeutic choices. The present issues with biomarker validation and application will also be highlighted in this review, along with suggestions for further study and clinical use [10]. In the end, this review will be an invaluable tool for medical professionals, researchers, and clinicians, helping them to better manage and treat patients with OSCC by utilising molecular biomarkers.

## 2 Diagnosis OF OSCC using molecular biomarkers

Identifying molecular biomarkers for early detection shows promise in enhancing outcomes for OSCC. Molecular biomarkers are biological substances present in blood, body fluids, or tissues that signal a regular or irregular process, state, or illness. Various types of biomarkers, such as genetic mutations, epigenetic alterations, protein levels, and circulating markers, have been studied for their possible diagnostic value in OSCC [11].

### 2.1 Genetic mutations

Genetic mutations in critical oncogenes and tumour suppressor genes play a crucial role in the development of OSCC. Mutations in the TP53 gene are one of the most researched genetic changes in OSCC. TP53, a gene that suppresses tumours, has a vital function in controlling the cell cycle, programmed cell death, and maintaining the stability of the genome. Around 60–70 % of OSCC cases show mutations in TP53, which frequently suggest a progression from a precancerous stage to invasive cancer. Identification of TP53 mutations using liquid biopsy techniques, like examining CtDNA, is becoming increasingly popular as a non-invasive method for diagnosis. Another important genetic indicator is the EGFR, which is highly expressed in a significant number of patients with OSCC. Overexpression of EGFR has been associated with a negative outlook, heightened tumour aggressiveness, and a lack of response to typical treatments [12]. Identifying EGFR mutations or amplifications in oral tissue biopsies or non-invasive samples, such as saliva, can serve as a significant biomarker for detecting OSCC at an early stage.

### 2.2 Epigenetic changes

Changes in DNA methylation, specifically epigenetic modifications, are known to be crucial in the progression of OSCC. Anomalous methylation of p16, DAPK1, and MGMT tumour suppressor genes has been detected in OSCC tissues and linked to early cancer development. MSP assays are in progress for identifying epigenetic changes in saliva and blood samples, providing a non-invasive method to pinpoint those at risk for OSCC. The excessive methylation of promoter areas in important genes such as *cyclin-dependent kinase inhibitor 2A* (CDKN2A) and *Death-associated protein kinase 1* (DAPK1) shows potential as a diagnostic biomarker, differentiating between cancerous and non-cancerous growths [13].

### 2.3 Protein-based biomarkers

Proteins implicated in the development, invasion, and metastasis of tumours can be crucial markers for OSCC diagnosis. The development of OSCC has been associated with the overexpression of several proteins, including Cyclin D1, MMP-9 and HIF-1α (*hypoxia-inducible factor 1-alpha*). The cell cycle regulator cyclin D1, which is linked to carcinogenesis and cell proliferation, is commonly overexpressed in OSCC. Similarly, MMP-9, which contributes to the extracellular matrix's breakdown, is linked to tumour invasion and metastasis and is frequently detected at high levels in OSCC [14]. These proteins offer non-invasive diagnostic alternatives since they can be found in bodily fluids like saliva or tissue samples.

### 2.4 Serological markers

Biomolecules called “serological markers” that are found in blood can reveal information about the onset, course, and response to therapy of cancer. The accuracy of diagnosis for oral squamous cell carcinoma (OSCC) is increased when many indicators are used. Numerous markers have been investigated for their diagnostic and prognostic value in OSCC, including *carcinoembryonic antigen* (CEA), *vascular endothelial growth factor* (VEGF), *cancer antigen* 125 (CA-125), *Cytokeratin 19 Fragment* (CYFRA 21-1), and *squamous cell carcinoma antigen* (SCCA). To summarise, although several markers such as VEGF, CA-125, CYFRA 21-1, SCCA, and CEA exhibit potential in the diagnosis and prognosis of OSCC, their unpredictability and overlap with other diseases demand a thorough, multimodal diagnostic approach as well as additional study to confirm their clinical suitability.

1. ***Carcinoembryonic antigen* (CEA)** is normally present in human serum at low levels—less than 5 ng/ml—but it can occasionally be raised in malignancies such as OSCC. Research indicates that OSCC patients had elevated levels of CEA in comparison to individuals with benign oral tumours or healthy controls, indicating the possibility of CEA serving as a biomarker. However, CEA is not employed as a standalone marker; rather, it is a component of a complete diagnostic approach because of its variability and rise in other disorders [15].
2. ***Vascular endothelial growth factor*,** or VEGF, plays a critical role in OSCC by encouraging angiogenesis, cancer cell invasion, and cell proliferation. In patients with OSCC, overexpression of VEGF has been linked to lymph node metastases, tumour development, and a poor prognosis. VEGF expression may play a part in tumour aggressiveness as it is also associated with radioresistance [16].
3. ***Cancer antigen* 125 (CA-125)** is higher in OSCC but is mostly linked to ovarian tumours. Research has indicated elevated levels of CA-125 in the saliva of individuals with OSCC, indicating its possible use as a salivary biomarker. Nonetheless, additional investigation is required to confirm its diagnostic and prognostic significance [17].
4. **CYFRA 21**–**1,** a fragment of *cytokeratin 19*, has been studied as a biomarker for various cancers, including OSCC. Elevated levels in saliva and serum of OSCC patients indicate its potential for detection. However, it lacks specificity to OSCC alone [18].
5. ***Squamous cell carcinoma antige*n (SCCA)**, a serine protease inhibitor, has been investigated for use in OSCC diagnosis, tracking, and prognosis. A positive prognosis is indicated by a decrease in SCCA levels after resection, but elevated levels may indicate the presence of SCC. However, because existing tests are unable to distinguish between its isoforms, contamination and detection limits remain [19].

### 2.5 Circulating tumour cells

Cancer metastasis is facilitated by *circulating tumour cells (*CTCs), which are cells that separate from a primary tumour and enter the bloodstream or lymphatic system. Nucleic acid amplification techniques such as PCR and immunocapture employing antibodies are approaches for identifying and evaluating CTCs. The CellSearch platform, approved by the FDA, identifies cells in blood that express cytokeratin or the epithelial cell adhesion molecule.CTCs aid in the development of novel treatments and offer insights into the biology of tumours. Their existence is associated with a worse prognosis and an increased likelihood of metastasis in cases of oral squamous cell carcinoma (OSCC). Certain markers, such as Ki-67, and genes linked to invasion and metastasis, including Twist1 or Snail, can identify CTCs and suggest a higher risk of relapse. Treatment failure may be caused by CTCs developing resistance to cancer therapy as a result of genetic and molecular alterations. Drug resistance may result from the heterogeneity of CTCs, hence understanding CTCs is crucial to creating countermeasures. Furthermore, CTC analysis aids in monitoring tumour response and assessing therapy efficacy; alterations in CTC quantity or features signify a favourable or unfavourable response, aiding in early identification and treatment modification decision-making [20].

### 2.6 SALIVARY biomarkers

More than 100 macromolecules, including DNA, RNA, and mRNA, as well as particular proteins like P53 and Cyfra 21-1, are salivary biomarkers. In particular, cytokines such as TNF-α and the interleukins IL-6 and IL-8 show great promise. Oral squamous cell carcinoma (OSCC) and other benign and malignant oral diseases are linked to elevated levels of these cytokines. These indicators are found in saliva using methods such as Luminex bead-based multiplex assays, Polymerase chain reaction (PCR), and enzyme-linked immunosorbent assays (ELISA). Research indicates that saliva from patients with OSCC has considerably greater levels of Interleukins (IL-6 and IL-8) than that of controls, indicating that these molecules may have use as diagnostic indicators. Saliva and serum both had comparable amounts of IL-8, but serum has larger levels of IL-6, indicating systemic involvement. *Tissue necrosis factor* (TNF-α) and other cytokines have demonstrated potential as indicators of precancerous lesions. Since OSCC is frequently detected at an advanced stage, these salivary biomarkers may be essential for early identification and better patient outcomes [21].

## 3 Treatment stratification OF OSCC using molecular biomarkers

The prevalent and aggressive kind of oral cancer known as oral squamous cell carcinoma (OSCC) varies greatly in how well people respond to treatment. By directing individualised treatment approaches, the use of biomarkers in treatment stratification has grown in significance in terms of improving patient outcomes. Because biomarkers can forecast how a patient will react to immunotherapy, radiation, and chemotherapy, more individualised and efficient treatment plans are made possible. This essay addresses developing biomarkers for immunotherapy in OSCC, the function of targeted medicines, and predictive biomarkers for response to radiation and chemotherapy.

### 3.1 Predictive biomarkers for chemotherapy and radiotherapy responses

Chemotherapy and radiotherapy remain cornerstone treatments for OSCC. However, not all patients respond equally, which underscores the need for predictive biomarkers to guide treatment decisions [22].

1. **Predictive Biomarkers for Chemotherapy Response**: Biomarkers that Predict Chemotherapy ResponseIt has been possible to predict the response of OSCC patients to chemotherapy using a number of biomarkers. The TP53 gene is one of the well-known biomarkers. In OSCC, TP53 mutations are common and linked to a poor prognosis as well as treatment resistance. Reduced susceptibility to cisplatin, a popular chemotherapeutic drug, has been associated with elevated levels of p53 protein production, which is a result of TP53 mutations. The biomarker *Epithelial Growth Factor Receptor* (EGFR) is another significant one. In OSCC, EGFR overexpression is commonly seen and linked to chemoresistant cells. Targeting high EGFR expression can be therapeutically beneficial since it can be used as a prediction of poor response to chemotherapy. Additionally, the effectiveness of 5-fluorouracil (5-FU), another chemotherapeutic medication used in the treatment of OSCC, has been linked to *human equilibrative nucleoside transporter 1* (hENT1) levels. Reduced medication absorption and, thus, decreased therapeutic efficacy can result from low hENT1 expression [[23], [24], [25], [26]].
2. **Predictive Biomarkers for Radiotherapy Response:** For OSCC, radiotherapy is frequently utilised as the main or adjunctive treatment. Patients who are most likely to benefit from radiation therapy can be identified with the use of predictive biomarkers for radiotherapy response [27]. EGFR is one such biomarker. EGFR overexpression has been linked to radiation resistance, much as its function in chemotherapy resistance. This is because EGFR plays a part in both cellular growth and DNA repair processes.An additional significant predictor of radiation response is HPV status. While oropharyngeal cancers are more frequently linked to HPV, its prevalence in OSCC has been investigated. In general, HPV-positive tumours respond to radiation therapy better than HPV-negative tumours [28]. Another important element influencing the results of radiation therapy is tumour hypoxia. Because hypoxia in tumours reduces oxygen availability, which is necessary for the production of reactive oxygen species that damage DNA, hypoxia can make radiation therapy less effective. Tumor hypoxia is measured and the response to radiation therapy is predicted by biomarkers such as carbonic anhydrase IX (CA IX) and hypoxia-inducible factor-1α (HIF-1α) [29].

### 3.2 Targeted therapies and the role of biomarkers in treatment selection

Targeted medicines have completely changed the way that many malignancies, including OSCC, are treated. The efficacy of these treatments, which target particular molecular targets linked to the development of cancer, can be greatly impacted by biomarkers.

1. **EGFR Inhibitors:** Treatment for OSCC using EGFR inhibitors, including cetuximab, has shown promise. The effectiveness of EGFR signalling biomarkers, such as KRAS mutations and EGFR expression levels, is vital in assessing the effectiveness of these inhibitors. Better responses to EGFR-targeted treatments are typically predicted by high EGFR expression and KRAS wild-type status [30].
2. **Inhibitors of VEGF:** Tumour angiogenesis is the aim of *vascular endothelial growth factor* (VEGF) inhibitors like bevacizumab. Biomarkers like VEGFR status and VEGF expression levels aid in the identification of patients who have a higher chance of responding well to these treatments. A poor prognosis is frequently linked to high VEGF expression in tumours, which may suggest a benefit from VEGF inhibitors [31].
3. **MEK and BRAF Inhibitors**: In certain cases of OSCC patients, *B-Raf proto-oncogene, serine/threonine kinase* (BRAF) and *Mitogen-activated protein kinase kinase* (MEK) inhibitors may be useful in treating a subgroup of patients who have particular genetic alterations. The patients who may benefit from these targeted medicines are identified using biomarkers for BRAF mutations. One important sign that BRAF inhibitors should be used is the existence of BRAF V600E mutations [32,33].

### 3.3 New biomarkers for OSCC immunotherapy

Immunotherapy has become a viable therapeutic option for a number of malignancies, including OSCC. Optimising treatment regimens requires the identification of appropriate biomarkers for predicting response to immunotherapy.

1. **Expression of PD-L1:** An established biomarker for predicting the response to immune checkpoint inhibitors, such pembrolizumab and nivolumab, is the expression of *programmed death-ligand 1* (PD-L1). One is more likely to respond to PD-1/PD-L1 inhibitors if tumour cells of immune cells in the tumour microenvironment display high levels of PD-L1 [34,35].
***2. Tumour Mutations Burden* (TMB):** The quantity of mutations found in a tumour's DNA is referred to as its tumour mutational load. Since tumours with greater mutation rates are more likely to display immune-recognizable neoantigens, high TMB has been linked to improved responses to immune checkpoint Inhibitors [36,37]. TMB evaluations can be used to determine which patients will benefit most from immunotherapy.
3. **Instability of Microsatellites (MSI):** Defects in DNA mismatch repair lead to microsatellite instability, which is another significant biomarker for immunotherapy. Since tumours with high MSI are more likely to develop neoantigens and have higher levels of immune cell infiltration, high MSI is suggestive of possible response to immune checkpoint inhibitors [38].
***4. Tumour Infiltrating Lymphocytes* (TILs)**: TILs, or *tumour-infiltrating lymphocytes*, Tumour -infiltrating lymphocyte composition and presence are becoming known as possible indicators for predicting immunotherapy responses. Higher TIL counts, especially those of CD8^+^ cytotoxic T cells, are typically linked to improved immunotherapy outcomes [39].

### 3.4 Current trends and future challenges in biomarkers for oral squamous cell carcinoma (OSCC)

One of the biggest developments in OSCC treatment has been the move towards personalised medicine. Biomarkers are essential for customising treatments for each patient. For example, the identification of particular genetic mutations and expression profiles has led to the development of more sophisticated targeted medicines [40]. Nowadays, the usage of EGFR inhibitors is determined by the presence of either high EGFR expression or receptor mutations; same methods are employed for other biological targets. This customisation aids in determining the best course of action, reducing unneeded side effects, and enhancing results overall. The comprehension of OSCC has been revolutionised by the progress made in genomic and molecular technology. Next-generation sequencing (NGS), a high-throughput sequencing method, enables thorough profiling of genomic abnormalities, including as mutations, copy number variations, and changes in gene expression. As a result, new biomarkers have been found, and multi-omic techniques that incorporate transcriptomic, proteomic, and genomic data have been developed [41]. A tendency towards using molecular data to inform treatment decisions can be seen in the rise of biomarkers for immunotherapy, such as microsatellite instability (MSI) and tumour mutational burden (TMB). With immune checkpoint inhibitors like pembrolizumab and nivolumab demonstrating efficacy in patients with high PD-L1 expression or TMB, immunotherapy has emerged as a promising treatment option for OSCC. The growing knowledge of the immune milieu surrounding tumours and the discovery of response-predictive biomarkers are driving the move towards immunotherapy [42]. Furthermore, combination medicines are being investigated to improve efficacy and overcome resistance, such as immune checkpoint inhibitors paired with chemotherapy or targeted therapies. In the field of biomarker research, liquid biopsies are an innovative approach. They provide a non-invasive substitute for conventional tissue biopsies in the areas of disease progression monitoring, treatment response evaluation, and minimal residual disease detection. Real-time insights into tumour dynamics and genetic mutations are being sought after through techniques like *circulating tumour DNA* (ctDNA) and circulating tumour cells (CTCs) [43]. Patients who are not suitable candidates for conventional biopsies can benefit greatly from liquid biopsies, which are also helpful for tracking treatment-related changes over time. The validation and standardisation of biomarkers remain a serious difficulty notwithstanding the advances made in this area. While many biomarkers exhibit promise in research environments, they are not validated or repeatable in clinical settings. It is crucial to guarantee that biomarkers are dependable, replicable, and applicable to various patient populations [44]. Furthermore, thorough validation through extensive, multi-center research is necessary for the incorporation of novel biomarkers into clinical guidelines. Patients with OSCC have varied genetic, epigenetic, and molecular profiles, making it a heterogeneous disease. This heterogeneity makes it difficult to find biomarkers that are applicable to all situations. Biomarkers that are successful for one patient subgroup might not be for another. Improving treatment outcomes requires developing techniques to deal with this variability, such as combining various biomarkers and stratifying patients based on molecular profiles. One major difficulty that persists is resistance to therapy, which includes targeted therapies, radiation, and chemotherapy. The complicated mechanisms that underlie resistance encompass a number of elements, including as the tumour microenvironment, altered signalling pathways, and genetic changes. Improving therapeutic efficacy requires an understanding of these mechanisms as well as the identification of biomarkers that either predict or aid in the overcome of resistance.Targeted medicines and improved biomarker testing provide substantial obstacles in terms of cost and accessibility. The availability of innovative medicines, liquid biopsies, and genomic sequencing may be restricted due to their high costs, especially in places where resources are limited [45]. In order to solve these problems, technological improvements must not only save prices but also guarantee that these diagnostic instruments and treatments are accessible to all.

## 4 Conclusion

A revolutionary development in oncology, the use of biomarkers into the therapy of oral squamous cell carcinoma (OSCC) allows for more individualised and efficient treatment plans. Present patterns emphasise the move towards personalised medicine, where immunotherapy, radiation, and targeted therapies are chosen based on biomarkers. Novel biomarkers, like tumour mutational burden (TMB) and PD-L1 expression, have been identified thanks to developments in genomic and molecular profiling. These biomarkers are essential for forecasting responses to immunotherapy. Liquid biopsies also provide a non-invasive way to track the development of a disease and the effectiveness of a treatment, improving patient care even more. But there are still a lot of obstacles to overcome. Biomarkers must be validated and standardised among a variety of patient populations in order for their clinical usefulness and dependability to be guaranteed. The development of biomarkers is complicated by the heterogeneity of OSCC, requiring methods that take patient genetic and molecular diversity into consideration. The complicated mechanisms underlying treatment resistance necessitate further research to find biomarkers that can either predict or lessen resistance. In addition, obstacles such as the high expense and restricted availability of targeted medicines and sophisticated diagnostic tools must be removed to guarantee fair patient access. It will take coordinated efforts in policy-making, technology development, and research to address these issues. Treatment accuracy for OSCC will be improved by ongoing technological and biomarker innovation combined with strong clinical validation. The evolution of personalised medicine in OSCC requires addressing ethical and regulatory issues as well as ensuring fair access to these developments. Overcoming these obstacles could lead to better patient outcomes and more tailored, efficient therapeutic approaches in the future of OSCC treatment.

## Ethical approval/patient consent

This is not applicable please include a comment indicating that no ethical approvals or patient consent were necessary for the study.

## Declaration of competing interest

The authors declare that they have no known competing financial interests or personal relationships that could have appeared to influence the work reported in this paper.
